# Acetylcholinesterase of the sand fly, *Phlebotomus papatasi* (Scopoli): construction, expression and biochemical properties of the G119S orthologous mutant

**DOI:** 10.1186/s13071-014-0577-4

**Published:** 2014-12-10

**Authors:** Kevin B Temeyer, Fan Tong, Maxim M Totrov, Alexander P Tuckow, Qiao-hong Chen, Paul R Carlier, Adalberto A Pérez de León, Jeffrey R Bloomquist

**Affiliations:** Agricultural Research Service, U. S. Department of Agriculture, Knipling-Bushland U.S. Livestock Insects Research Laboratory, 2700 Fredericksburg Road, Kerrville, TX 78028-9184 USA; Department of Entomology and Nematology, Emerging Pathogens Institute, University of Florida, 2055 Mowry Road, PO Box 100009, Gainesville, FL 32610-00009 USA; Molsoft LLC, 3366 North Torrey Pines Court, Suite 300, La Jolla, CA 92037 USA; Department of Chemistry, Virginia Tech, 900 West Campus Drive, 480 Davidson Hall, Blacksburg, VA 24061-0001 USA

**Keywords:** Sand fly, Acetylcholinesterase inhibition, *P. papatasi*, cDNA, AChE

## Abstract

**Background:**

*Phlebotomus papatasi* vectors zoonotic cutaneous leishmaniasis. Previous expression of recombinant *P. papatasi* acetylcholinesterase (PpAChE1) revealed 85% amino acid sequence identity to mosquito AChE and identified synthetic carbamates that effectively inhibited PpAChE1 with improved specificity for arthropod AChEs compared to mammalian AChEs. We hypothesized that the G119S mutation causing high level resistance to organophosphate insecticides in mosquitoes may occur in PpAChE1 and may reduce sensitivity to inhibition. We report construction, expression, and biochemical properties of rPpAChE1 containing the G119S orthologous mutation.

**Methods:**

Targeted mutagenesis introduced the G119S orthologous substitution in PpAChE1 cDNA. Recombinant PpAChE1 enzymes containing or lacking the G119S mutation were expressed in the baculoviral system. Biochemical assays were conducted to determine altered catalytic properties and inhibitor sensitivity resulting from the G119S substitution. A molecular homology model was constructed to examine the modeled structural interference with docking of inhibitors of different classes. Genetic tests were conducted to determine if the G119S orthologous codon existed in polymorphic form in a laboratory colony of *P. papatasi*.

**Results:**

Recombinant PpAChE1 containing the G119S substitution exhibited altered biochemical properties, and reduced inhibition by compounds that bind to the acylation site on the enzyme (with the exception of eserine). Less resistance was directed against bivalent or peripheral site inhibitors, in good agreement with modeled inhibitor docking. Eserine appeared to be a special case capable of inhibition in the absence of covalent binding at the acylation site. Genetic tests did not detect the G119S mutation in a laboratory colony of *P. papatasi* but did reveal that the G119S codon existed in polymorphic form (GGA + GGC).

**Conclusions:**

The finding of G119S codon polymorphism in a laboratory colony of *P. papatasi* suggests that a single nucleotide transversion (GGC → AGC) may readily occur, causing rapid development of resistance to organophosphate and phenyl-substituted carbamate insecticides under strong selection. Careful management of pesticide use in IPM programs is important to prevent or mitigate development and fixation of the G119S mutation in susceptible pest populations. Availability of recombinant AChEs enables identification of novel inhibitory ligands with improved efficacy and specificity for AChEs of arthropod pests.

## Background

Leishmaniasis is a widespread debilitating and neglected disease of intertropical and temperate regions affecting millions of people throughout the world. The most common form is cutaneous leishmaniasis, with an estimated 0.7 to 1.3 million new cases annually, caused by flagellated protozoans in the genus *Leishmania* transmitted by the bite of several sand fly species [1-3]. *Leishmania major* is the predominant pathogen of zoonotic cutaneous leishmaniasis that is vectored (transmitted) in the Middle East, Asia, Africa and Southern Europe by *Phlebotomus papatasi* (Scopoli) [4-6]. The vector of cutaneous leishmaniasis, *P. papatasi*, impacted U.S. military readiness and operations in Iraq and Afghanistan [7-10], and the ability to control *P. papatasi* is important to millions of people in endemic areas of the world. The primary means to control zoonotic leishmaniasis transmission is through reduction of rodent habitat or rodent treatment to reduce local sand fly populations and the use of chemical insecticides and insecticide-treated bednets to reduce human bites by sand flies [2,11-17]. Organophosphate and carbamate insecticides may be used for control of insect vectors of infectious disease, acting through the inhibition of acetylcholinesterase in the central nervous system. We previously reported genetic and biochemical properties of recombinant acetylcholinesterase (AChE) of *P. papatasi*(rPpAChE1), and noted that PpAChE1 had 85% amino acid sequence identity to AChEs of *Culex pipiens* and *Aedes aegypti* mosquito species [18]. Point mutations resulting in production of an altered, insensitive AChE comprise a major mechanism of resistance to organophosphate and carbamate insecticides [19-21], and preliminary evidence of organophosphate resistance has been reported in sand flies [22-24]. It was previously hypothesized that the major mutation responsible for high level resistance to organophosphate inhibition in mosquito AChE (G119S, *Torpedo* AChE nomenclature [25]) [26-28] may occur in *P. papatasi* [18]. Here, we report the construction, baculoviral expression, and biochemical properties of recombinant PpAChE1 (rPpAChE1) containing the G119S orthologous mutation.

## Methods

### Targeted mutagenesis and baculoviral expression of rPpAChE1-G119S

A baculovirus expression vector containing the cDNA encoding PpAChE1 [18] was used as the template for targeted mutagenesis. A serine codon (AGC) was substituted for the glycine codon (GGA) at nucleotide positions 837-839[GenBank: JQ922267] to generate the G119S orthologous mutation (*Torpedo* AChE nomenclature) in PpAChE1 cDNA. Essentially, high-fidelity PCR utilized phosphorylated primers (SigmaGenosys, St. Louis, MO) PpAChE768U25-GGC (5′Phos-CTTCTACTCAGGAACATCCACACTC-3′) and PpAChE748L20-OPR (5′Phos-CTACCACCGAAGATCCATAG-3′) with Phusion HotStart DNA polymerase (New England BioLabs, Ipswich, MA) and template DNA (pBlueBac4.5/V5-His containing PpAChE1 coding sequence [18]) preincubated at 98°C for 30 sec followed by 25 cycles of 10 sec at 98°C, 45 sec at 65°C, and 5 min at 72°C with a final 10 min incubation at 72°C. The amplified product was ligated using a Quick Ligation™ Kit (New England BioLabs) according to the manufacturer’s instructions, transformed into chemically competent TOP10 *E. coli* cells (Life Technologies, Carlsbad, CA) and plated onto L-agar plates containing 100 μg/ml carbenicillin (Sigma Chemical Co, St. Louis, MO). Transformant colonies were selected, plasmid DNA sequenced to verify correct construction of the PpAChE1 containing the G119S orthologous mutation, cotransfected with Bac-N-Blue DNA into Sf21 insect cell culture for baculovirus expression, and initially characterized in microplates using a modified Ellman’s assay as described previously [18].

### Sand flies, RNA, cDNA synthesis, and agarose gel electrophoresis

Sand flies used in this study were from a laboratory colony of *P. papatasi* maintained at the USDA-ARS, Knipling-Bushland U.S. Livestock Insects Research Laboratory in Kerrville, Texas. Sand fly colony derivation, maintenance, preparation of RNA, cDNA synthesis and agarose gel electrophoresis were as previously described [18].

### Anticholinesterases as probes of enzyme function

**Figure 1:**
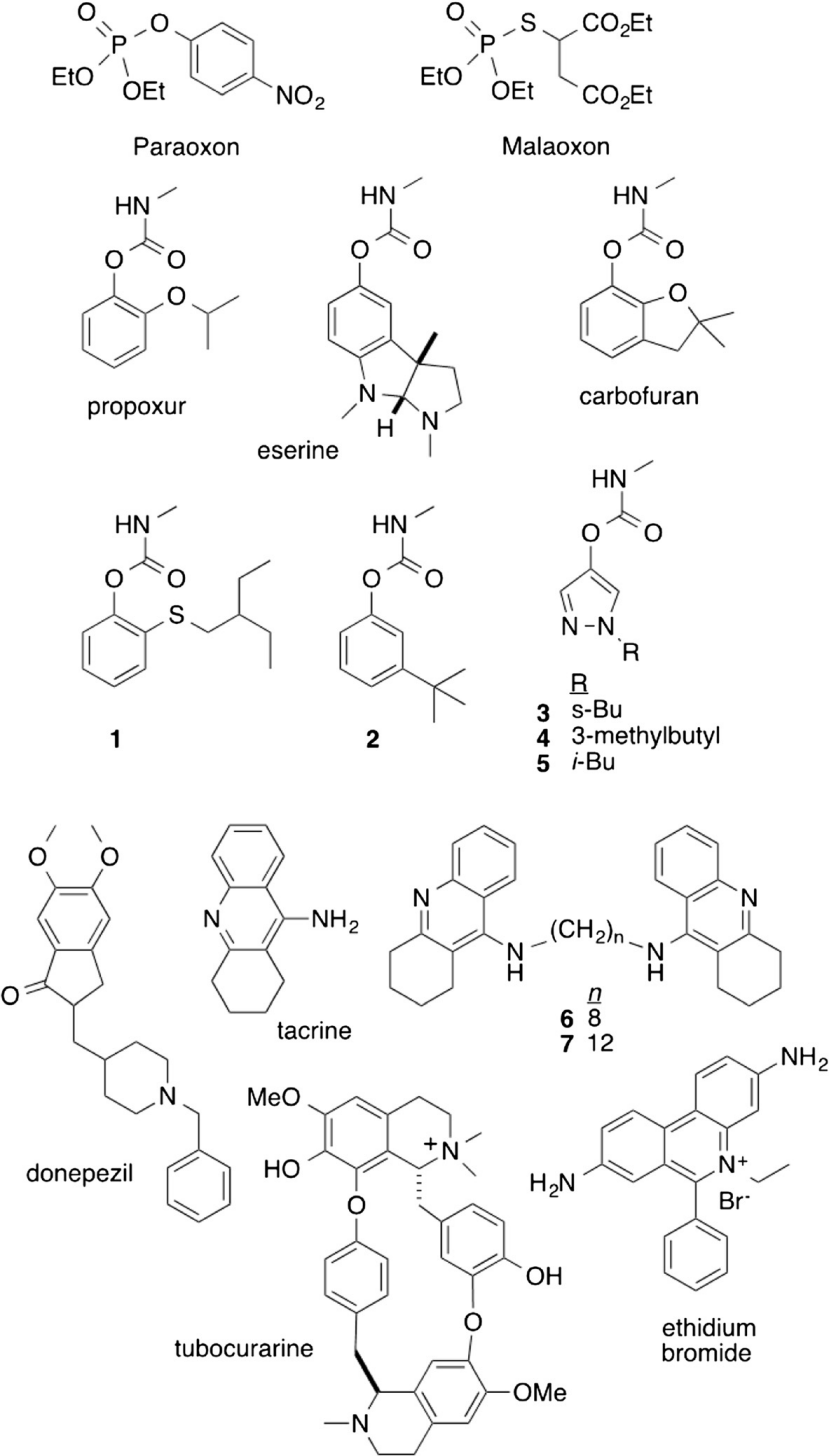
**Chemical structures and names of experimental anticholinesterases used in this study.** Bold numbers beside the names denote the compounds as presented in the text. For the *bis*(n)-tacrines, “n” refers to the number of methylene groups in the linker. Each compound was assigned to an inhibitor class as given in Table 1.

The experimental anticholinesterases used in this study for enzyme characterization are shown in Figure 1. They were synthesized and purified via established methods [29-31] and had purities of at least 95%. The synthesized experimental carbamates were as follows: **1**, 2-((2-ethylbutyl)thio)phenyl methylcarbamate; **2**, 3-(*tert*-butyl)phenyl methylcarbamate; **3**, 1-(*sec*- butyl)-1*H*-pyrazol-4-yl methylcarbamate; **4**, 1-isopentyl-1*H*-pyrazole-4-yl methylcarbamate; **5**, 1-isobutyl-1*H*-pyrazol-4-yl methylcarbamate; **6**, *N*^1^,*N*^6^-bis(1,2,3,4-tetrahydroacridin-9-yl)hexane-1,6-diamine; and **7**, *N*^1^,*N*^7^-bis(1,2,3,4-tetrahydroacridin-9-yl)heptane-1,7-diamine. In addition, a range of commercially available AChE inhibitors were purchased. The inhibitors eserine (99% pure), propoxur (99%), carbofuran (99%), donepezil (98%), tacrine (99%), and ethidium bromide (95%) were all purchased from Sigma–Aldrich (St. Louis, MO, USA). D-Tubocurarine (99%) was obtained from Alfa Aesar (Ward Hill, MA, USA).

### Biochemical characterization and inhibition assays

In this study, three categories of AChE inhibitors were chosen to define the pharmacological profiles of wild type and G119S rPpAChEs. They included catalytic site inhibitors (organophosphates, carbamates, tacrine, and eserine), peripheral site inhibitors (tubocurarine and ethidium bromide), and bivalent inhibitors (*bis*(8)-tacrine, *bis*(12)-tacrine, and donepezil). Note that tacrine differs from the other catalytic site inhibitors in that it is reversible, and does not covalently bind the catalytic serine. Tacrine binds in the choline-binding site, and does not extend into the oxyanion hole or acyl pocket [32]. The compounds were made into stock solutions by dissolving in DMSO, and all enzyme assays were run in constant 0.1% DMSO as a carrier. Inhibition of rPpAChE by these inhibitors was determined using the Ellman assay in a 96-well plate configuration [33]. The rPpAChE cell lysates were pre-incubated with at least six concentrations of inhibitors for 30 minutes at room temperature prior to adding 300 μM 5,5′-dithiobis-(2-nitrobenzoic acid) (DTNB) and 400 μM acetylthiocholine enzyme substrate (AcSCh), which were both dissolved in 0.1 M sodium phosphate buffer, pH 7.0. The kinetic reading of absorbance at 405 nm was started immediately after adding DTNB and AcSCh with a Dynex Triad multimode plate reader (Dynex Technologies, Chantilly, VA, USA). Inhibitor concentration-response curves and inhibition parameters were constructed by nonlinear regression to a four parameter logistic equation using GraphPad Prism 4.0c software (GraphPad Software, San Diego, CA, USA).

### Construction of a ligand docking molecular homology model of PpAChE1

A molecular homology model of *P. papatasi* AChE1 (wild type) was built in ICM [34] by homology [35] based on a 2.6 Å resolution mouse AChE X-ray structure, Protein Data Base code 4B84 [36]. The template enzyme has 48% overall identity with the target sequence. Local homology in the active site region was significantly stronger. Seven tightly bound water molecules in the vicinity of the active site in the template structure were transferred into the model and their positions were refined by energy optimization (water molecules number 46, 49, 52, 55, 71, 72 and 146). The G119S mutation (position 256 in PpAChE1 sequence, GenBank: AFP20868.1) was next introduced into the model (in ICM). After optimization of the side chain conformation within the otherwise rigid protein, residual clashes of S256 with F425 and Y258 (*P. papatasi* numbering) were detected. The F425 clash was relieved by relaxation of its side-chain, while the Y258 clash could not be relieved by side chain relaxation alone but was resolved after backbone relaxation within the G255-S259 residue window (*i.e.*, a loop including ±1 residue around S256 and Y258 each). Relaxation resulted in 1.1 Å /0.6 Å RMSD displacement of, respectively, all heavy atoms/only backbone atoms within this region. Docking of representative ligands was performed in ICM Docking module [37,38]. For ligands with a covalent inhibition mechanism (carbamates), the tetrahedral transition state on the reaction pathway between non-covalently bound inhibitor and acylated enzyme was modeled, using ‘covalent docking’ protocol in ICM [34]. Because observed AChE ligand-bound conformations often vary in the sidechain conformation of residue F/Y330 (*T. californica* numbering) in the active site gorge, a multiple receptor conformation ‘4D docking’ approach [39] was applied to sample two rotamers of Y465 (*P. papatasi* numbering). Three lowest-scoring conformations were retained in each docking simulation, visually inspected and compared to available X-ray structures of the same or similar ligands bound to AChE (of other species such as mouse and *T. californica).* The final models chosen were either the lowest or second-lowest conformation (the latter was selected if it was in a significantly better agreement with experimentally observed interaction modes). To identify potentially adverse interactions caused by the G119S (*Torpedo californica* numbering) mutation, docked ligand/PpAChE (wt) complexes were superimposed with PpAChE1-G119S model and superimposed structures were analyzed for ligand/PpAChE-G119S clashes.

### Test for G119S codon sequence in *P. papatasi* laboratory colony PpAChE1

The PCR-RFLP assay of Weill et al. [28] was modified to test for the presence of the G119S orthologous mutation in our laboratory colony of *P. papatasi*. A segment of *P. papatasi* genomic or cDNA was amplified by PCR using primers PpAChE-793U17 (5′-CCACGTCCCAAAAACTC-3′) and PpAChE-842 L23 (5′-GAGTGTGGATGTTCCTGAGTAGA-3′) and the 72 bp amplicon was tested for the presence of the G119S orthologous codon by incubation with Alu I restriction endonuclease (New England BioLabs) followed by gel electrophoresis. Positive (G119S orthologous rPpAChE1*,* this report) and negative (wild type rPpAChE1, [18]) control templates were used to validate the assay. If the G119S orthologous codon was present in the template, Alu I digestion resulted in cleavage of the DNA amplicon to 25 bp and 47 bp segments. A similar PCR-RFLP test was used to test for sequence polymorphisms (GGA vs GGC) in the G119S orthologous codon, using PCR primers PpAChE-814U26AluC (5′-GTTATGCTATGGATCTTCGGTGGTAG-3′) and PpAChE-854 L22 (5′-TCGTACACATCGAGTGTGGATG-3′). Alu I digestion of the 54 bp amplicon produced 28 bp +36 bp fragments if position 839 [GenBank: JQ922267] was the C nucleotide. Positive and negative control templates were used to validate the assay.

## Results

### Targeted mutagenesis and baculoviral expression of rPpAChE1-G119S

**Figure 2:**
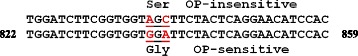
**Partial PpAChE1 cDNA sequence containing G256S codon identified as OP-R in** ***An. gambiae*** **(G119S) and other insects.** Numbers designate beginning and ending nucleotide sequence numbers from GenBank JQ922267.1.

**Figure 3:**
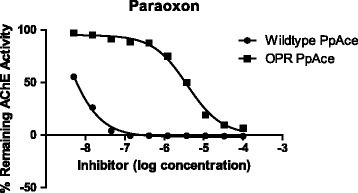
**Relative sensitivity of the altered rPpAChE1-G119S enzyme to paraoxon inhibition was reduced by over 1000-fold as a result of the single amino acid substitution.**

The rPpAChE1 constructed by targeted mutagenesis (rPpAChE1-G119S) was completely sequenced and verified to contain the G119S orthologous codon (AGC) at nucleotide positions 837–839 [GenBank: JQ922267] of rPpAChE1 cDNA (Figure 2). As shown in Figure 3, the single amino acid substitution (G119S) in rPpAChE1-G119S resulted in greater than 1000-fold reduction in sensitivity to paraoxon inhibition compared to rPpAChE1 (wild type). The G119S orthologous substitution also exhibited a 4-fold increased *K*_m_ (Michaelis-Menten constant; *i.e*., concentration of substrate producing ½ maximal velocity) for the substrate acetylthiocholine (AcSCh), where the *K*_m_ (μM) values were 24 and 98 for rPpAChE1and rPpAChE1-G119S, respectively.

### Biochemical characterization and inhibition assays

As shown in Figure 3, paraoxon was a potent inhibitor of wild type enzyme (rPpAChE1), but not rPpAChE1-G119S. The other anticholinesterases (Figure 1) demonstrated a wide range of potencies as well as resistance ratios for the inhibition of both strains of rPpAChE (Table 1). The calculated IC_50_ values and confidence limits had correlation coefficients, R^2^, of at least 0.95, except those curves with very wide confidence limits due to the high resistance of the G119S rPpAChE to OPs and carbamates. For wild type rPpAChE, all of the catalytic site inhibitors and bivalent inhibitors showed moderate to high potencies to inhibit enzyme activity, with IC_50_ values from the middle nanomolar (e.g., propoxur and paraoxon) to sub nanomolar concentrations (compound **7**), although most compounds fell in the range of 3–76 nM (Table 1). On the other hand, the two peripheral site inhibitors had low potencies for rPpAChE inhibition of 17 μM (ethidium bromide) and 143 μM (tubocurarine) analogous to similarly low affinity of the peripheral site inhibitor propidium for mammalian AChE [40].

**Table 1:** **Inhibition of rPpAChE1 and rPpAChE1-G119S by different classes of AChE inhibitors**

**Compound**	**Inhibitor class**	**Wild type r** ***Pp*** **AChE1**	**G119S r** ***Pp*** **AChE1**	**Resistance Ratio**
		^**a**^ **IC**_**50**_ **(95% CI)**	^**a**^ **IC**_**50**_ **(95% CI)**	
Paraoxon	acylation site	2.863 (1.862–4.401)	3,819 (3,205–4,550)	1336
Malaoxon	acylation site	4.361 (3.184–5.972)	1,972 (1,665–2,340)	452
Eserine	acylation site	3.2 (2.6–4.0)	86 (73–102)	27
Propoxur	acylation site	220 (147–329)	4,227,000 (−−)^b^	19,213
Carbofuran	acylation site	24 (17–33)	124,000 (−−)	5,200
**1**	acylation site	14 (10–19)	236,800 (7,404–7,575,000)	17,000
**2**	acylation site	36 (28–48)	123,100 (−−)	3,400
**3**	acylation site	13 (9.4–19)	235 (164–336)	18
**4**	acylation site	75 (36–152)	4,775 (3,048–7,482)	64
**5**	acylation site	76 (50–117)	2,128 (1,267–3,573)	28
Tacrine	choline binding site	67 (56–81)	388 (318–473)	5.8
**6**	bivalent	0.42 (0.35–0.52)	2.7 (2.3–3.2)	6.4
**7**	bivalent	14 (13–15)	35 (28–42)	2.5
Donepezil	bivalent	52 (39–70)	262 (202–341)	5.0
Tubocurarine	peripheral site	143,200 (94,630–216,700)	661,800 (290,000–1,511,000)	4.6
Ethidium Bromide	peripheral site	17,100 (13,890–21,060)	6,433 (4,380–9,448)	0.4

^a^IC50 = inhibitor concentration producing 50% inhibition of activity (in nM), where (95% CI) = 95% confidence interval.

^b^(−−) denotes wide confidence limits from incomplete inhibition of rPpAChE1-G119S.

In contrast, the G119S rPpAChE showed strong resistance to the organophosphates (paraoxon and malaoxon) and all phenyl-substituted methylcarbamates (compounds **1**, **2**) with resistance ratios over 450. Interestingly, a group of alkyl-substituted pyrazole carbamates (compounds **3**, **4**, and **5**), which include a smaller ring than phenyl methylcarbamates, had much lower resistance ratios (18–64 fold) compared to phenyl methylcarbamates (Table 1). All other peripheral site inhibitors, bivalent inhibitors, and a catalytic site inhibitor, tacrine, showed the lowest resistance ratios, which were ≤7.

An exception was eserine, which despite having a large pyrroloindole ring system, displayed much less cross resistance than the phenylcarbamates, but a bit more than tacrine and the bivalent inhibitors (Table 1). Current data with wild type rPpAChE showed good correlation to that previously published for 11 compounds (eserine, propoxur, carbofuran, tacrine, *d*-tubocurarine, ethidium bromide, donepezil, **1**, **2**, **6**, and **7**), which differed only in that a shorter 10 min preincubation with inhibitor was used [29]. The data sets collected in both studies for rPpAChE were not normally distributed (D’Agostino & Pearson omnibus normality test), but were highly correlated with nonparametric Spearman r = 0.884 (0.59-0.97; 95% CL) and two-tailed P < 0.0006.

### Inhibitor docking in a molecular homology model of PpAChE1

**Figure 4:**
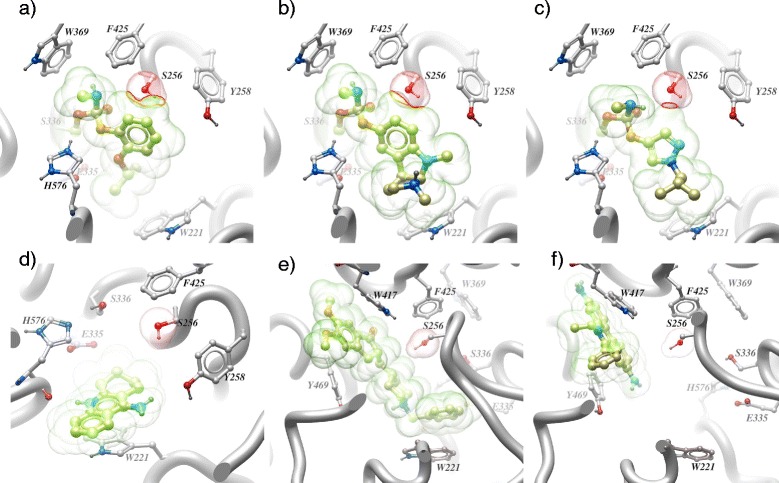
**Representative inhibitors (from Table** 1 **) docked into PpAChE1 (wild type) and superimposed into the G119S (PpAChE position S256) mutant: 4a) propoxur, a phenylcarbamate; 4b) eserine, a cationic carbamate; 4c) pyrazolecarbamate (compound 4), a ‘small core’ carbamate; 4d) tacrine, a non-covalent active site inhibitor; 4e) donepezil, a bivalent inhibitor; 4f) ethidium, a peripheral site inhibitor.** Van der Waals surfaces of the inhibitor (green) and mutated serine S256 (PpAChE1 sequence numbering) hydroxyl (red) are shown. Red contour lines delineate the overlap (clash) region (where present). Key residues such as W221 (active site ‘floor’), W417 (peripheral site wall), catalytic serine S336 and histidine H576, as well as sidechains F425 and Y258 most affected by the mutation at G/S256 are shown in ball-and-stick representation.

A molecular homology model of PpAChE1 (Figure 4) was constructed based on murine AChE. Selected inhibitors were docked into the model which was then adjusted for the G119S (*T. californica* numbering) mutation at PpAChE1 position 256. As shown in Figure 4, propoxur (4a) docked into the molecular homology model exhibits a fairly large region of Van der Waals overlap, suggesting that the G119S mutation (S256 in the model) results in a large interference with propoxur docking, in agreement with the results presented in Table 1 (resistance ratio 19,213). Eserine (4b) appears to exhibit a similarly large region of Van der Waals overlap, suggesting that it should also exhibit a significantly high resistance ratio in the G119S mutant; however, the experimentally measured resistance ratio (Table 1) is only 27. Compound **4** (4c) exhibits a significantly reduced Van der Waals overlap, in relative agreement with the measured resistance ratio of only 64. Tacrine (4d) is not directly impacted by the G119S substitution, but may be somewhat affectedby desolvation of the catalytic serine (S336) exhibiting a resistance ratio of only 5.8. Donepezil (4e) also shows no direct impact with the G119S substitution and provides a minimal resistance ratio of only 5. Similarly, ethidium (4f) exhibits no interaction with the G119S substitution (S256) and exhibits a resistance ratio of 0.4.

### Test for G119S codon sequence in *P. papatasi* laboratory colony PpAChE1

**Figure 5:**
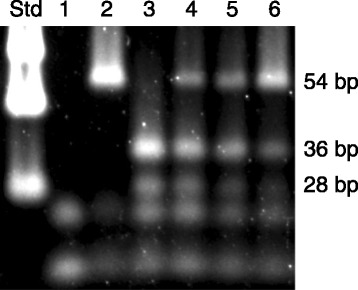
**PCR-RFLP assay for polymorphism in laboratory** ***P. papatasi*** **.** Template DNA (as indicated for each lane) was amplified by PCR then subjected to digestion with Alu I and electrophoretically separated on a 4% Metaphor agarose gel. Lane: Std, DNA size standards; 1, no template negative control; 2, wild type PpAChE1 plasmid; 3, PpAChE1-G119S plasmid; 4–6, genomic DNA extracted from individual *P. papatasi* colony females fed sugar water only, no blood.

The PCR-RFLP assay adapted from Weill et al. [28] failed to demonstrate the presence of the G119S orthologous mutation in our laboratory colony of *P. papatasi*; however, direct sequencing of a small percentage of cDNA clones that included the codon corresponding to the G119S orthologous site in *PpAChE1* and a PCR-RFLP assay designed to detect the presence of a GGC codon at nucleotide positions 837–839 [GenBank: JQ922267] both indicated the presence of polymorphic GGC/GGA sequence at the codon position orthologous to the G119S mutation in mosquitoes (Figure 5). Preliminary data suggests that the GGC codon at this locus is present in our laboratory flies at an estimated frequency between 10-20%.

## Discussion

The G119S mutation of rPpAChE has significant effects on the catalytic properties and inhibitor sensitivity of the enzyme. The four-fold increase seen in *K*_m_ is similar to the two-fold increase in *K*_m_ seen in the G119S mutant of *Anopheles gambiae* AChE [30]. Furthermore, high enzyme resistance ratios are seen for aryl methylcarbamate (*e.g*., propoxur, carbofuran), as was seen for AgAChE-G119S [30,41]. High resistance ratios are also seen for paraoxon and malaoxon. Like the aryl methycarbamates, these compounds acylate the active site serine (acylation site inhibitors) and extend into the oxyanion hole, where G119 is located. In contrast, the pyrazol-4-yl methylcarbamates (Table 1, compounds **3**–**5**) possess significantly smaller insensitivity ratios, as we previously observed for AgAChE-G119S [30]. The smaller volume of pyrazol-4-yl core inhibitors (Figure 1, compounds **3**–**5**) relative to aryl methylcarbamates presumably allows them to effectively enter the crowded active sites of G119S mutant *Anopheles gambiae* AChE and rPpAChE1-G119S. Tacrine is also a catalytic site inhibitor, but unlike carbamates and organophosphates, binds in the choline-binding site, rather than the oxyanion hole. Thus, tacrine inhibition is largely unaffected by the G119S mutation and the resistance ratio is only 5.8 (Table 1). Similarly low resistance ratios are seen for bivalent inhibitors (compounds **6**,**7**, and donepezil) and peripheral site inhibitors. Since neither class of inhibitor bind AChE near G119S, the mutation does not affect inhibition by these compounds.

The molecular homology model docking of selected inhibitors (Figure 4) is in good general agreement with the measured resistance ratios of selected inhibitors. The G119S mutation (S256) in the PpAChE1 model (Figure 4) interferes with positioning of phenylcarbamates for acylation transition state, while for non-covalent inhibitors there are no direct steric issues. Desolvation of serine OH might explain small residual resistance. The exception is the docking model for eserine (Figure 4b), which is a special case among carbamates because as a bulky cationic lipophilic moiety, it may function as a non-covalent inhibitor even if acylation (*i.e.*, covalent inhibition mechanism) is impaired by the mutation. For the three carbamates, docking was done assuming a covalent inhibition mechanism (actual models are of the acylation transition state). If eserine is modeled non-covalently, it could still have hydrophobic and cation-pi interactions at least as extensive as tacrine, therefore this discrepancy may not have so much to do with homology model accuracy as with more complex mechanistic issues.

In summary, results indicate that the single amino acid substitution orthologous to the G119S mutation responsible for high level resistance to organophosphate and carbamate insecticides in mosquitoes can also generate high level resistance to inhibition by acylation site inhibitors in recombinant *P. papatasi* AChE1. The recent reports of aryl methylcarbamates that were shown to have improved targeting of pest AChEs relative to mammalian AChEs [41,42], suggests that use of the recombinant enzymes with various amino acid substitutions may offer platforms for SAR modeling and *in vitro* screening to design and identify novel inhibitors with specific targeting of insecticide-insensitive AChEs that also exhibit improved mammalian safety profile. Further studies are planned or underway to evaluate the effects of additional mutations in PpAChE1, to evaluate the presence of G119S orthologous codon polymorphism in natural populations of *P. papatasi*, to evaluate additional synthetic ligands to assess their efficacy against wild type and “mutant” forms of rPpAChE1, and to utilize molecular modeling and structure activity relationships (SARs) to improve construction and selection of inhibitory lead chemical structures.

In mosquitoes, the G119S substitution produces high level organophosphate and carbamate insecticide resistance but also a high fitness cost (in the absence of insecticide) when homozygous [43-45], presumably due to 30-fold reduction in turnover number for substrate and approximately 70% decrease in cholinergic activity [46]. Reduction in G119S allele frequency was reported in Lebanon over a 3–4 year period presumably resulting from switching to pyrethroids for mosquito control and loss of the G119S allele due to fitness cost in the absence of inhibitor selection pressure [47]. In spite of the fitness cost, the G119S-containing *ace-1* allele is widespread throughout the world [48] and the fitness cost may be reduced in the presence of *kdr*-resistance to pyrethroids [49] or by duplication of the *ace-1* allele to permit maintenance of a heterozygous state, essentially fixing it in the population [50-52]. Agricultural pesticide use and mosquito control efforts have largely resulted in the spread of the *ace-1* duplication in West Africa [53]. Together, these findings provide strong warnings about the need for careful use of insecticides that provide strong selection for resistance to organophosphates and carbamates. Once the G119S substitution occurs, pyrethroid use may allow reduction of the frequency of the G119S allele [47], or selection for *kdr*-based resistance to pyrethroids may result in multiple-resistant pest populations by reducing fitness cost of the G119S allele [49]. The finding of G119S orthologous codon polymorphism in a laboratory colony of *P. papatasi* strongly suggests that a single nucleotide transversion (GGC → AGC) might readily occur, causing relatively rapid development of resistance to organophosphate insecticides if subjected to strong selection. Careful management of pesticide use in IPM programs is important to prevent or mitigate development and fixation of the G119S mutation in susceptible pest populations. Availability of the recombinant AChEs may enable identification of novel inhibitory ligands with improved efficacy and specificity for AChEs of arthropod pests.

## Conclusions

We demonstrated that the G119S orthologous substitution in PpAChE1 produces high levels of resistance to OP and carbamate inhibitors, suggesting a strong likelihood of resistance development if the subject codon is polymorphic (GGA + GGC) in natural populations of *P. papatasi*. PCR and sequencing tests indicate that the G119S orthologous codon is polymorphic (GGA or GGC) in our laboratory *P. papatasi* colony. We are currently seeking *P. papatasi* specimens from natural populations worldwide to determine if the G119S orthologous codon is polymorphic in natural populations.

As noted by Weill *et al*., “The development of new insecticides that can specifically inhibit the G119S mutant form of acetylcholinesterase-1 will be crucial in overcoming the spread of resistance” [26]. Use of the recombinant *P. papatasi* AChE1 and revised molecular models may facilitate rapid screening *in silico* and *in vitro* to identify novel PpAChE1 inhibitor ligands, and comparative studies on biochemical kinetics of inhibition. Construction and expression of mutant forms of PpAChE1 will facilitate the development of rapid molecular assays and other tools to screen and characterize mutations giving rise to organophosphate-insensitive PpAChE1. Addition of new molecular data on PpAChE1may also be used in modeling studies to predict *in vivo* insecticidal activity for novel inhibitors as described by Naik *et al*. [54]. Availability of the recombinant PpAChE1 will enable the creation of mechanism-based screens to discover more effective inhibitors that may be developed to innovate safer vector control technologies.

## Endnotes

^a^This article reports the results of research only. Mention of a proprietary product does not constitute an endorsement by the USDA for its use.

^b^USDA is an equal opportunity provider and employer.

^c^Copyright statement: Copyright protection is not available for any work of the United States Government.

^d^Disclaimer: "The views expressed in this article are those of the authors and do not necessarily reflect the official policy or position of the Department of Agriculture, Department of Defense, nor the U.S Government”.

## Abbreviations

AChE: Acetylcholinesterase
DTNB: Dithiobisnitrobenzoic acid
AcSCh: Acetylthiocholine
BuSCh: Butyrylthiocholine
rPpAChE1: Recombinant *P. papatasi* AChE1
